# Emergence and dissemination of an IncC plasmid co-harbouring *bla*_KPC-2_, *bla*_NDM-1_ and *bla*_VIM-24_ carbapenemase genes across multiple Gram-negative species in Colombia

**DOI:** 10.1093/jacamr/dlag222

**Published:** 2026-10-16

**Authors:** Roberto Sierra, Betsy Castro, Elsa De La Cadena, Laura J Rojas, Juan Carlos García-Betancur, Mélanie Roch, Steven M Marshall, Nalleth Bolaño-Ardila, Susan D Rudin, Janneth J Escobar-Arcos, Clara J Salazar-Montes, Belkis Palacio-Villalba, Elizabeth Paz, Robert A Bonomo, María Virginia Villegas, Diego O Andrey

**Affiliations:** Division of Laboratory Medicine, Department of Diagnostics, Geneva University Hospitals, Geneva, Switzerland; Department of Microbiology and Molecular Medicine, University of Geneva, Geneva, Switzerland; Grupo de Investigaciones en Resistencia Antimicrobiana y Epidemiología Hospitalaria (RAEH), Universidad El Bosque, Bogotá, Colombia; Grupo de Investigaciones en Resistencia Antimicrobiana y Epidemiología Hospitalaria (RAEH), Universidad El Bosque, Bogotá, Colombia; Department of Molecular Biology and Microbiology, Case Western Reserve University School of Medicine, Cleveland, OH, USA; CWRU-Cleveland VAMC Center for Antimicrobial Resistance and Epidemiology (Case VA CARES) Cleveland, OH, USA; Grupo de Investigaciones en Resistencia Antimicrobiana y Epidemiología Hospitalaria (RAEH), Universidad El Bosque, Bogotá, Colombia; Department of Microbiology and Molecular Medicine, University of Geneva, Geneva, Switzerland; Research Service, Louis Stokes Cleveland Department of Veterans Affairs Medical Center, VA Northeast Ohio Healthcare System, Cleveland, OH, USA; Departamento de Microbiología, Universidad Popular del Cesar, Valledupar, Cesar, Colombia; Facultad de Medicina, Doctorado en Medicina Tropical, Universidad de Cartagena, Cartagena, Colombia; Research Service, Louis Stokes Cleveland Department of Veterans Affairs Medical Center, VA Northeast Ohio Healthcare System, Cleveland, OH, USA; Department of Medicine, Case Western Reserve University School of Medicine, Cleveland, OH, USA; Laboratorio Clínico, Clínica Imbanaco, Grupo Quirónsalud, Cali, Colombia; Laboratorio de Microbiología, Clínica Nuestra Cali, Cali, Colombia; Fatoxacol SAS. Departamento de Investigación, Grupo: FATOXACOL, Cartagena, Colombia; Universidad de Cartagena, Departamento de Farmacología, Cartagena, Colombia; Comité de Infecciones, Hospital Departamental Tomas Uribe Uribe, Tuluá, Valle del Cauca, Colombia; Department of Molecular Biology and Microbiology, Case Western Reserve University School of Medicine, Cleveland, OH, USA; CWRU-Cleveland VAMC Center for Antimicrobial Resistance and Epidemiology (Case VA CARES) Cleveland, OH, USA; Research Service, Louis Stokes Cleveland Department of Veterans Affairs Medical Center, VA Northeast Ohio Healthcare System, Cleveland, OH, USA; Department of Medicine, Case Western Reserve University School of Medicine, Cleveland, OH, USA; Departments Pharmacology, Biochemistry and Proteomics and Bioinformatics, Case Western Reserve University School of Medicine, Cleveland, OH, USA; Grupo de Investigaciones en Resistencia Antimicrobiana y Epidemiología Hospitalaria (RAEH), Universidad El Bosque, Bogotá, Colombia; Comité de Infecciones y Vigilancia Epidemiológica, Clínica Imbanaco, Grupo Quirónsalud, Cali, Colombia; Division of Laboratory Medicine, Department of Diagnostics, Geneva University Hospitals, Geneva, Switzerland; Department of Microbiology and Molecular Medicine, University of Geneva, Geneva, Switzerland; Infectious Diseases Division, Department of Medicine, Geneva University Hospitals and Medical School, Geneva, Switzerland

## Abstract

**Objectives:**

Carbapenem-resistant Gram-negative bacilli (CRGNB) pose a public health threat, increasingly driven by plasmid-mediated dissemination of carbapenemase genes. As part of a Colombian genomic monitoring programme, we aimed to characterize CRGNB co-harbouring multiple carbapenemase genes and determine their genetic context.

**Methods:**

We screened 832 carbapenem-resistant isolates collected during 2011–2023 using carbapenemase-targeted qPCR. Ten isolates co-harbouring *bla*_KPC_, *bla*_NDM_ and *bla*_VIM_ underwent antimicrobial susceptibility testing, long-read whole-genome sequencing, comparative genomics and conjugation assays.

**Results:**

Nine Enterobacterales isolates and one *Aeromonas caviae* isolate from unrelated patients in geographically distant Colombian hospitals carried closely related IncC plasmids ranging from 160 to 317 kb. These plasmids co-harboured *bla*_KPC-2_, *bla*_NDM-1_ and *bla*_VIM-24_ within three conserved multidrug resistance regions, including a Tn*21*-associated region carrying *bla*_NDM-1_ and *bla*_VIM-24_ and a Tn*6454*-associated *bla*_KPC-2_ platform, alongside class 1 integrons, insertion sequences and multiple additional antimicrobial resistance determinants. The ten isolates were resistant to multiple β-lactam/β-lactamase inhibitor combinations, and five were cefiderocol resistant. Aztreonam/avibactam remained active against all isolates; colistin resistance was confined to intrinsically resistant *Providencia rettgeri* and *Serratia sarumanii*. Conjugation assays demonstrated plasmid transfer to *Escherichia coli* J53, and sequencing confirmed transfer of a 179 kb IncC plasmid with functional conjugation machinery. Comparative genomic analysis showed a highly conserved IncC plasmid backbone across multiple species, supporting broad dissemination of this resistance platform.

**Conclusions:**

This first report of a single plasmid co-harbouring three carbapenemase genes. This finding highlights a concerning mechanism for interspecies dissemination of MDR Gram-negative pathogens and underscores the importance of long-read genomic surveillance focused on high-risk plasmids.

## Introduction

Carbapenem-resistant Gram-negative bacilli (CRGNB) represent a significant global health threat due to limited treatment options available for these multidrug-resistant pathogens.^1^ Carbapenemases, including serine carbapenemases (*e.g.* KPC, OXA-48) and metallo-β-lactamases (MBL; *e.g.* NDM, VIM), have disseminated globally.^2^ Carbapenemase genes disseminate through successful bacterial lineages and mobile genetic elements (MGEs), such as plasmids and transposons. KPC-2 was first reported in 2005 in Colombia, where KPC is endemic, followed by VIM (2011) and NDM (2013).^3,4^  *bla*_KPC-2_ remains the most prevalent carbapenemase gene among *K. pneumoniae* isolates, particularly within high-risk clones ST307 and ST1082, while *bla*_KPC-3_ is strongly associated with ST11.^5^ These clones commonly harbour a diversity of plasmids, such as IncC and IncF replicon types.^5^

Sporadic CRGNB co-producing dual carbapenemases have been reported for more than a decade in the USA, Europe and Latin America,^2,6–9^ these combinations most commonly involving a serine carbapenemase together with a MBL, thereby broadening resistance to β-lactam due to their distinct susceptibility profiles to β-lactamase inhibitors. Of particular concern, in Colombia, the proportion of Enterobacterales co-harbouring multiple carbapenemases increased markedly, rising from 1.1% in 2018 to nearly 50% of tested isolates in 2024 co-producing KPC and NDM.^10,11^ The introduction of ceftazidime/avibactam and meropenem/vaborbactam has expanded treatment options for serine β-lactamase producers, although the increasing prevalence of isolates co-harbouring a MBL threatens the effectiveness of these therapies, substantially limiting therapeutic options.^6^

The limited information available on the genetic elements mobilizing multiple carbapenemase genes hinders our understanding of their dissemination. Long-read sequencing enables reconstruction of the genomic context of resistance determinants within a strain, identifying whether they are carried on separate plasmids, as is often the case, or co-localized on the same genetic element.^12^ In this study, leveraging a large national genomic monitoring collection of clinically relevant CRGNB, we identified clinical isolates co-producing multiple carbapenemases and characterized a novel conjugative IncC plasmid co-harbouring *bla*_KPC-2_, *bla*_NDM-1_ and *bla*_VIM-24_ across multiple Gram-negative species recovered from Colombian hospitals.

## Materials and methods

Within the *Grupo de Investigación en Resistencia Antimicrobiana y Epidemiología Hospitalaria* (RAEH), 832 clinical or carriage CRGNB isolates collected during 2011–2023 from 37 hospitals across 17 Colombian cities were screened for carbapenemase genes by *in-house* qPCR.^13^ Ten isolates co-harbouring three distinct carbapenemases, confirmed with the NG-test^®^ CARBA5 immunoassay (NG Biotech, France), were identified and retained for this study. MICs were determined using Sensititre EUMDRXXF panels (ThermoFisher Scientific, USA) and UMIC cefiderocol strips (cat. no. UM-CID-040, Bruker Daltonics, Germany), and by gradient diffusion for aztreonam/avibactam (cat. no. 92089, Liofilchem, Italy), both ranging between 0.03–32 mg/L. Testing and result interpretation followed EUCAST V15.0 guidelines and breakpoints, including reference strain ATCC 25922. Conjugation assays were performed as previously described^12^ between *K. pneumoniae* UEB72 (the earliest isolate) and *E. coli* J53, selecting on meropenem (1 mg/L). High-molecular weight DNA (Promega, USA) was extracted from exponential-phase broth cultures as previously described^12^ and used for WGS of the ten clinical isolates and transconjugant using long-read sequencing (Oxford Nanopore Technologies, UK) with the Sequencing by Ligation kit V14 and R10.4.1 flow cells. Assembly was performed using Flye (v2.9.4), followed by polishing with Medaka (v1.12.1) and annotation with Center for Genomic Epidemiology databases and tools (MLST v2.0.9, ResFinder v2.4.0, PlasmidFinder v2.2.0) and MOB-Suite (V3.1.9). The cgMLST analysis was performed using Ridom SeqShere+ (v10.5.0). Plasmid alignments and regions were visualized with ProkSee (build 2026–02–25).

## Results

Ten isolates consistently carrying *bla*_KPC-2_, *bla*_NDM-1_ and *bla*_VIM-24_ were recovered from adult patients in hospitals across Colombia, with the earliest isolate dating back to 2019 (Tables S1–S2, available as Supplementary data at *JAC-AMR* Online). These isolates included five *K. pneumoniae* (Kpn), one *K. aerogenes* (Kae), one *Enterobacter hormaechei* (Eho), one *Providencia rettgeri* (Pre) and one *Serratia sarumanii* (Ssa; *S. marcescens* complex) associated with clinical infection, as well as one *Aeromonas caviae* (Acv) recovered from rectal screening. Genomic analysis showed that the *K. pneumoniae* isolates belonged to distinct genetic lineages: ST987 (Kpn_UEB72), ST17 (Kpn_UEB62), ST1441 (Kpn_UEB50) and ST307 (Kpn_UEB85 and Kpn_UEB87, differing by 11 loci based on cgMLST) (Figure 1; Table S1).

**Figure 1.**
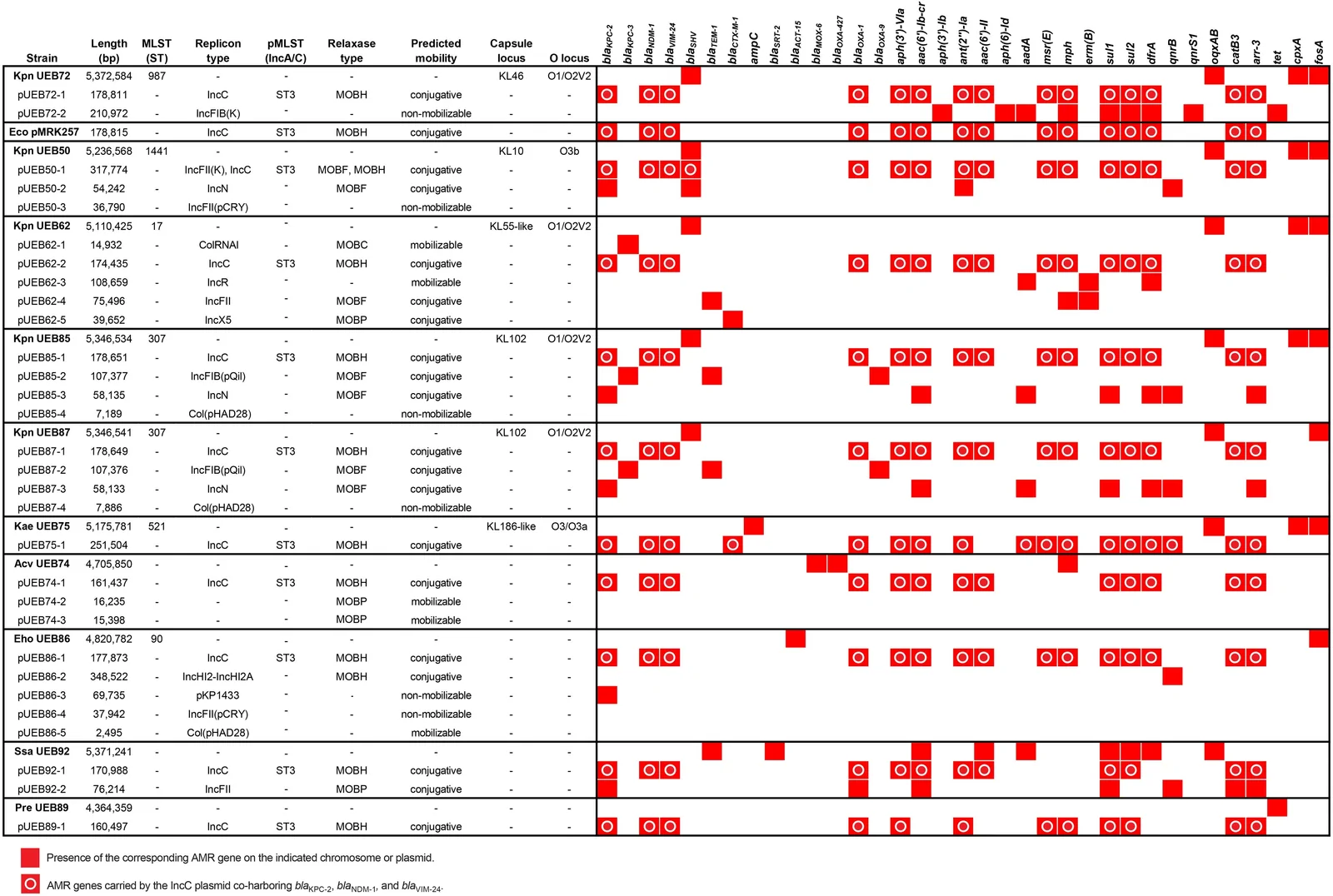
Genomic characteristics and antimicrobial resistance determinants of clinical isolates co-harbouring *bla*_KPC-2_, *bla*_NDM-1_ and *bla*_VIM-24_. Clinical isolates and associated plasmids are grouped by strain. Columns indicate chromosome or plasmid length, MLST, plasmid replicon type, relaxase type, predicted mobility, capsule locus (KL), O locus and the presence of antimicrobial resistance genes. Squares indicate the gene presence, with circles identifying resistance genes carried by the IncC plasmid. The conserved IncC plasmids carrying *bla*_KPC-2_, *bla*_NDM-1_ and *bla*_VIM-24_ are highlighted by shared resistance profiles and conjugative MOBH relaxases across multiple bacterial species. Additional plasmid and resistance determinants identified in individual isolates are also shown. MRK257 corresponds to the *E. coli* J53 transconjugant obtained from conjugation assays with Kpn_UEB72.

All isolates exhibited an MDR phenotype, with broad resistance to β-lactams, including the newer β-lactam/β-lactamase inhibitor (BL/BLI) combinations ceftazidime/avibactam, imipenem/relebactam and meropenem/vaborbactam, while remaining susceptible to aztreonam/avibactam (Figure 1; Table S1). Five isolates, three *K. pneumoniae* (UEB72, UEB50 and UEB62), *P. rettgeri* (UEB89) and *S. sarumanii* (UEB92), were also resistant to cefiderocol. All isolates remained susceptible to colistin, except the intrinsically resistant *P. rettgeri* and *S. sarumanii*. Complete antimicrobial susceptibility profiles are shown in Table S1.

WGS enabled comparative genomic analysis of the genetic context associated with triple carbapenemase production. All ten genomes harboured a highly conserved IncC plasmid backbone carrying *bla*_KPC-2_, *bla*_NDM-1_ and *bla*_VIM-24_. Most IncC plasmid sizes ranged from 160 to 179 kb except for a 251 kb in Kae_UEB75 and a 317 kb multireplicon plasmid in Kpn_UEB50 (Figure 1). Using the 178 kb plasmid Kpn_UEB72, recovered from the earliest isolate, as a reference, we observed a highly conserved structure across all isolates (Figure 2). Three multidrug resistance regions (MDRr), containing clustered AMR genes and MGEs, were identified. MDRr1 contained a Tn*21*-associated region with four modules: (i) a class 1 integron (*dfrA17*, *aac(6’)-Ib-cr*, *bla*_OXA-1_, *catB3*, *arr-3*), (ii) *bla*_NDM-1_ flanked by IS*91* and IS*4321*, (iii) another class 1 integron (*bla*_VIM-24_, *aac(6’)-II*, *catB3*, *ant(2'’)-Ia*, *aph(2'’)-VIa* and *sul1)* and (iv) IS*1353* and the mercury resistance operon *merEDCPTR.* MDRr2 contained a Tn*6454*-associated *bla*_KPC-2_ platform with Δ*bla*_TEM_, *tnpR* and *tnpA* genes. MDRr3 harboured arsenic, macrolide (*msr*(E), *mph*(E) and *ermD*) and sulphonamide (*sul2*) resistance determinants flanked by IS*6* and IS*91* (Figure 2).

**Figure 2.**
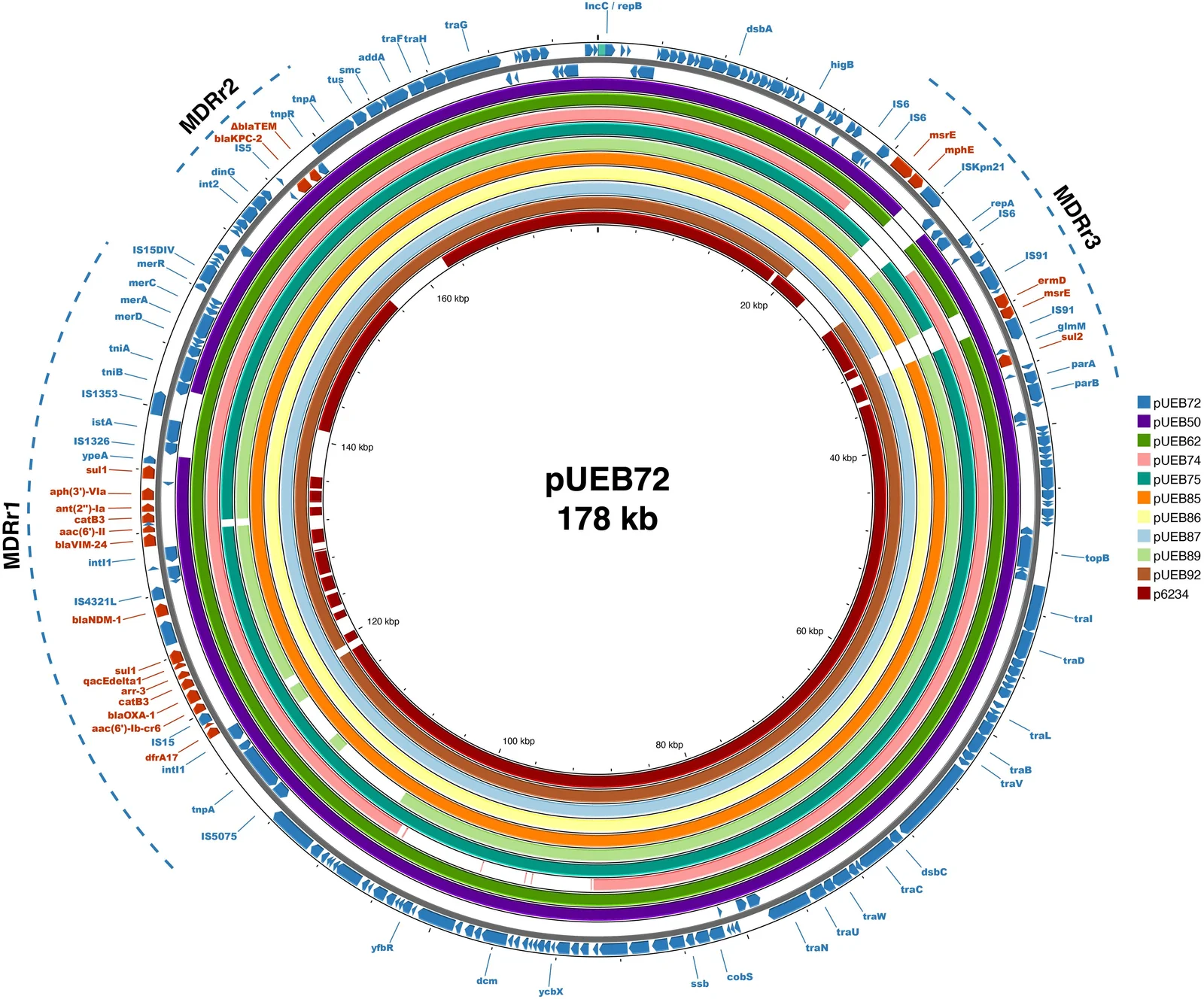
Comparative analysis of IncC plasmids co-harbouring *bla*_KPC-2_, *bla*_NDM-1_ and *bla*_VIM-24_ in multiple CRGNB species. Circular comparative alignment of IncC plasmids identified in ten isolates representing different Gram-negative bacterial species, using pUEB72 (178 kb) as the reference plasmid. Sequence comparisons and annotations were visualized using ProkSee. Concentric rings represent individual plasmids, with sequence similarity to pUEB72 indicated by shading and gaps representing non-homologous regions. The previously reported Colombian plasmid p6234, which carries *bla*_NDM-1_ as its sole carbapenemase gene, is included for comparison.^14^ The outer annotation track displays predicted coding sequences and key genetic features, including antimicrobial resistance genes, insertion sequences and transposition-associated genes, conjugation-associated genes (*tra*), integrases and plasmid backbone genes. Three conserved multidrug resistance regions (MDRr1–3) are indicated, including a Tn*21*-associated region carrying *bla*_NDM-1_ and *bla*_VIM-24_, and a Tn*6454*-associated *bla*_KPC-2_ platform, and MDRr3, carrying macrolide and sulphonamide resistance determinants.

In the conjugation assay, *K. pneumoniae* UEB72 successfully transferred the plasmid to *E. coli* J53 (conjugation frequency: 4.4 × 10^−3^), and WGS performed on the transconjugant confirmed the presence of a 179 kb IncC plasmid co-harbouring the three carbapenemase genes along with transfer-associated features including *oriT*, the MOBH relaxase, type IV coupling proteins, a type IV secretion system and the *higBA* toxin-antitoxin system for plasmid stability (Figures 1 and 2).

## Discussion

We have identified an emerging high-risk plasmid in Colombia by screening a large national CRGNB genomic monitoring collection. Our analysis revealed a novel IncC plasmid co-harbouring *bla*_KPC-2_, *bla*_NDM-1_ and *bla*_VIM-24_ genes in six different species, isolated from geographically distant Colombian hospitals. Its detection in healthcare settings across the country without any clear spatiotemporal link (except for the two closely related ST307 isolates) suggests broad and successful dissemination. Our results indicate that this plasmid is broad-host-range and self-conjugative. It harboured distinct multidrug resistance regions, including a Tn*21*-associated region carrying both *bla*_NDM-1_ and *bla*_VIM-24_, and a non-Tn*4401 bla*_KPC-2_ platform (Tn*6454*). To our knowledge, the co-localization of these three carbapenemase genes on a single plasmid has not been previously reported.

The combination of *bla*_NDM-1_*, bla*_KPC-2_ and *bla*_VIM-24_ on a single plasmid is consistent with the molecular epidemiology in Colombia, where (i) KPC is endemic, (ii) MBLs are increasingly reported and (iii) IncC plasmids are widely associated with antimicrobial resistance in GNB.^14^ Globally, dual carbapenemase-producing isolates have been reported with increasing frequency. Between 2012 and 2019, the U.S. CDC recorded over 150 cases of Enterobacterales harbouring multiple carbapenemase genes, often linked to healthcare exposure abroad.^15^ However, these reports did not specify whether the genes were located on the same plasmid. In several countries, notably those with endemic carbapenemase circulation, multiple carbapenemase genes have been identified in the same strain, although typically located on separate genetic elements. Such cases have been reported in *K. pneumoniae* isolated from Greece, Colombia and elsewhere, often involving KPC, VIM and NDM on distinct plasmids or unresolved genetic platforms.^14,16,17^

In our study, three *K. pneumoniae*, *E. hormaechei* and *P. rettgeri* isolates also carried additional copies of *bla*_KPC_ (including *bla*_KPC-3_), while *A. caviae* harboured an extra copy of *bla*_VIM-24,_ located on separate plasmids or chromosome (Figure 1; Table S1). The co-localization of three carbapenemase genes on a single plasmid, as demonstrated in this study, represents yet another step in Gram-negative resistance evolution, significantly increasing the potential for plasmid-mediated gene transfer across bacterial species. Combinations of serine carbapenemases and MBLs pose a significant clinical challenge. While ceftazidime/avibactam and meropenem/vaborbactam are effective against KPC-producing Enterobacterales, their activity is compromised when MBLs are co-produced. Aztreonam/avibactam remained active against all ten isolates with low MICs; however, its limited availability in many low- and middle-income countries represents a significant issue. Colistin remained the only active option that is widely available but has limited clinical efficacy. Although five isolates were resistant to cefiderocol, this phenotype was not transferred by conjugation from *K. pneumoniae* UEB72 to *E. coli* J53, indicating that, at least in this isolate, it is not strictly plasmid encoded.

This study highlights the value of long-read sequencing for genomic surveillance of highly resistant CRGNB, enabling accurate reconstruction of complete plasmids and detection of co-located resistance genes, MGEs and plasmid backbones across diverse hosts. Such capabilities are critical for tracking and monitoring high-risk plasmids, such as the one described here. Further studies are warranted to elucidate the origin, transmission dynamics and persistence of this element. Continued genomic surveillance, infection control measures and antimicrobial stewardship programmes remain essential to curb the dissemination of these pathogens.

## Supplementary Material

dlag222_Supplementary_Data

## Data Availability

Data are available under BioProject No. PRJNA1292751.

## Supplementary data

Tables S1 to S2 are available as Supplementary data at *JAC-AMR* Online.
